# Salivary Proteins—A Barrier on Enamel Demineralization: An *in vitro* Study

**DOI:** 10.5005/jp-journals-10005-1398

**Published:** 2017-02-27

**Authors:** Mithra N Hegde, Ankit R Sajnani

**Affiliations:** 1 Head, Department of Conservative Dentistry and Endodontics AB Shetty Memorial Institute of Dental Sciences, Mangaluru Karnataka, India; 2 Postgraduate Student, Department of Conservative Dentistry and Endodontics AB Shetty Memorial Institute of Dental Sciences, Mangaluru Karnataka, India

**Keywords:** Albumin, Demineralization, Enamel, Salivary protein.

## Abstract

**Aim:**

The aim of this study is to evaluate the protective effect of the salivary proteins on the demineralization of enamel.

**Materials and methods:**

Twenty freshly extracted human molar teeth were used in this study. Enamel samples (2 mm thickness) were prepared from the buccal and lingual surfaces of the teeth selected. An acid-resistant nail varnish was used to cover every aspect of the sample, except an area of 5 * 5 mm limited by an adhesive tape. After drying, the adhesive tape was removed, exhibiting a rectangular area on the enamel surface. Samples were divided into two groups:

- *Group I (10 samples):* Each sample was coated by 100 μg of albumin for 2 hours at 37°C.
- *Group II (10samples):* Each sample was exposed to 100 μL of deionized water.

Samples were washed by dipping once in deionized water. They were then disposed into individual tubes containing demineralization solution for 1, 2, 3, and 4 minutes at 37°C with gentle agitation. The demineralization solution was utilized to determine the calcium loss from specimens at 1, 2, 3, 4 minutes using an ultraviolet-visible spectrophotometer.

**Results:**

Calcium loss was less from the albumin-coated samples than control group at all times and was statistically significant (p < 0.05). Also, calcium loss was maximum at the end of 1 minute, and it decreased as time interval increased and was statistically significant (p < 0.001).

**Conclusion:**

Albumin has provided a strong protection against enamel demineralization at all times compared to the one without it.

**How to cite this article:**

Hegde MN, Sajnani AR. Salivary Proteins—A Barrier on Enamel Demineralization: An *in vitro* Study. Int J Clin Pediatr Dent 2017;10(1):10-13.

## INTRODUCTION

In the oral cavity, saliva surrounds the tooth and creates the milieu that bathes the tooth surface. It serves as the main vehicle for solubilizing and transporting potential harmful substances as well as protective factors to the biofilm covered tooth surface. Saliva’s protective role is mediated by its ability to clear cariogenic food substances and dilute, neutralize, and buffer organic acids formed by biofilm microorganisms. Thus, it reduces the demineral ization rate and enhances remineralization by providing calcium, phosphate, and fluoride in the fluid phase of the biofilm in close association with the tooth surface.^1^

The selective adsorption of salivary proteins leads to formation of an organic film on the tooth surface, i.e., known as the acquired enamel pellicle (AEP).^2,3^ It forms a protective interface between the tooth surface and the oral environment and acts as a selective permeability barrier that regulates demineralization/rem-ineralization processes.^4^ Also, it acts as a reservoir of remineralizing electrolytes, i.e., calcium, phosphate, and fluorides.^1^

Organic phase of saliva consist of proteins that may regulate the demineralization and remineralization processes. Similar to saliva, AEP also consist of albumin-, mucin-, and proline-rich proteins (PRPs), histatins and cystatins which have shown high affinity to enamel surfaces and are also involved with the maintenance of tooth integrity by favoring a suitable calcium phosphate environment. Recently, by using the proteomic technology, more than 130 different proteins have been identified in the dental pellicle.^1^

Despite the variety of proteins, information regarding the importance of salivary proteins on demineralization/ remineralization processes is scarce.^5^

Hence, the study aimed to evaluate the protective effect of the salivary proteins on the demineralization of enamel.

## MATERIALS AND METHODS

### Infection Control Protocol

The teeth were cleansed of visible blood and gross debris and were maintained in a hydrated state during storage. Then they were placed in sodium hypochlorite solution diluted with saline in a ratio of 1:10 in the container with a secure lid to prevent leakage.^6^

Inclusion Criteria

Freshly extracted human maxillary and mandibular molar teeth extracted for periodontal problems. Teeth were selected based on randomized sampling method.

Exclusion Criteria

Teeth with caries, hypoplastic lesions, white spots, cracks, erosion, developmental anomaly, or any other deformity.

### Enamel Sample Preparation

Twenty freshly extracted human molar teeth were used in this study. Enamel samples (2 mm thickness) were prepared from the buccal and lingual surfaces of the teeth selected, using a double-faced diamond disk mounted on a contra-angle handpiece. An acid-resistant nail varnish was used to cover every aspect of the specimen, except an area of 5 × 5 mm limited by an adhesive tape. After drying, the adhesive tape was removed from the enamel using a sharp-tipped instrument exhibiting a rectangular area on the enamel surface.^7^

### Groups

A total of 20 enamel samples were divided into two groups of 10 samples in each.

- *Group 1*: Each sample was coated by 100 ug of albumin (bovine serum albumin was used as a standard model protein) for 2 hours at 37°C.
- *Group 2:* Each sample will be exposed to 100 uL of deionized water.

Samples were washed by dipping once in deionized water. They were then disposed in demineralization solution: Calcium chloride (CaCl_2_) - 2.2 mM, sodium dihydrogen phosphate (NaH_2_PO_4_) - 2.2 mM, acetic acid - 0.005M, pH 4.5 (Siqueira et al) for 1, 2, 3, and 4 minutes at 37°C with gentle agitation.

The demineralization solution was utilized to determine the calcium loss from samples at 1, 2, 3, and 4 minutes using an ultraviolet-visible light spectro-photometer.

**Graph 1:**
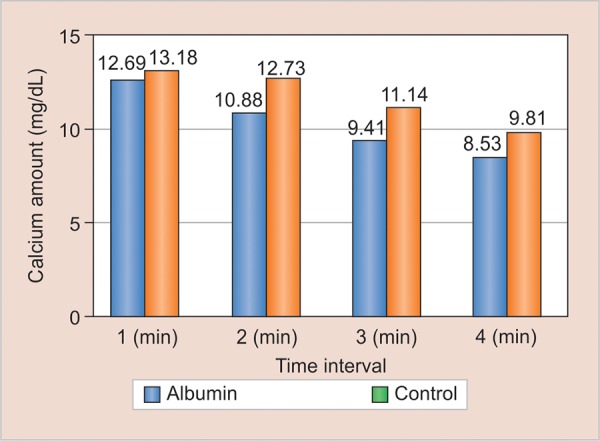
Comparison of calcium loss between study groups

**Graph 2:**
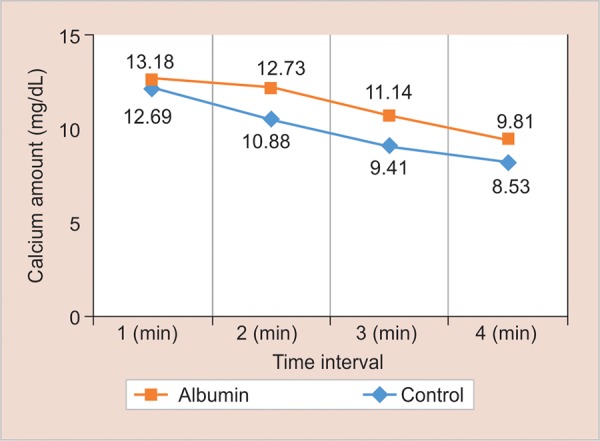
Comparison of calcium loss at different time intervals from the study groups

### Assessment of the Extent of Demineralization

Amount of Calcium released

The calcium content of the samples was analyzed using a quantitative colorimetric calcium determination assay (Agappe Calcium Assay Kit, Ernakulum, Kerala, India), employing an ultraviolet-visible spectrophotometer (Systronics, Ahmedabad, Gujarat, India) determining the optical density at a wavelength of 612 nm.

### Formula to calculate Calcium Loss

Amount of calcium loss(mg/dl) = (Optical density of test sample / Optical density of standard sample) × 10

Optical density of standard sample = 0.645

### Statistical Analysis

Results were analyzed using Independent Sample t-test and Repeated Measure ANOVA Test.

## RESULTS

Graph 1 shows that calcium loss was less from the albumin coated enamel samples than control group at all times. It was statistically significant (p < 0.05) except at the end of 1 minute.

Graph 2 shows that calcium loss was maximum at the end of 1 minute, and it decreased as time interval increased, i.e., at the end of 2, 3, and 4 minutes. Also, the amount of calcium loss at the end of 1 minute was more and statistically significant (p < 0.001) than at the end of 4 minutes.

## DISCUSSION

Saliva, a body fluid basically formed by proteins and ions, has multiple physiological functions, such as digestion, swallowing, lubrication, tooth integrity, and antimicrobial protection.^4^ A group of salivary proteins, namely, statherin, the acidic PRPs, albumin, histatins, and cystatins are also said to be multifunctional as they are partly responsible for the remineralization capacity of saliva.^8^

Albumin is the most abundant serum protein, accounting for more than 50% of all plasma proteins. In the oral cavity, albumin is considered as a serum ultrafiltrate to the mouth and is present in saliva due to contamination by either traces of blood or gingival fluid.^9^ It may also diffuse into the mucosal secretions. Concentration of salivary albumin varies considerably from person to person.^10^ As such there are no reference limits for salivary albumin, but it has shown to be increased in medically compromised patients, such as in immunosuppression, radiotherapy, and diabetes.^11^

In the present study enamel samples were coated with albumin for a period of 2 hours as there are several studies showing that AEP reaches a plateau within 2 hours on enamel.^12,13^ The loss of calcium from albumin coated enamel samples was less than the control group at all the time intervals, i.e., 1, 2, 3, and 4 minutes (Graph 1). This could be because enamel is mainly protected from demineralization by the inhibitory effects of salivary albumin which penetrates into the pores and binds to hydroxyapatite (HA) crystals in addition to the protective effects of AEP.^14,15^ These results were in accordance with the studies on other salivary proteins conducted by Kielbassa et al,^15^ Featherstone et al,^16^ Kosoric et al^17^ and Martins et al.^4^ Studies conducted by Hemingway et al^18^ has also shown that egg protein ovalbumin has potential antierosive properties and can be used as additive to drinks. Also, a study conducted by Hegde et al concluded that with the decrease in the levels of albumin there is increase in the levels of dental caries.^19^

In the present study, maximum calcium loss was seen from the enamel samples at the end of 1 minute from both albumin-coated enamel samples and control group, and it decreased as the time interval increased, i.e., 2, 3, and 4 minutes (Graph 2).

As stated earlier, saliva is a rich reservoir of calcium, phosphate, and fluoride ions. According to the law of saturation, a dynamic equilibrium exists between the mineral contents of the tooth and saliva. At “neutral pH,” the HA crystal dissolves minimally and releases the calcium, phosphate, and hydroxyl ions into saliva, but since it already contains the same minerals, saliva becomes supersaturated which results in the precipitation of these minerals back on to the tooth surface.^20^

Critical pH is a point at which a solution is just saturated with a mineral. It is inversely proportional to the amount of calcium and phosphate ions in the saliva of an individual, since the concentration of these ions vary from each individual, critical pH also changes. Research now suggests that in individuals with low calcium and phosphate ion concentration levels, the critical pH is 6.5, and those with higher concentration of these ions, critical pH is 5.5.^21^

At “acidic pH” (which in our study is 4.5), the phosphate ions and the hydroxyl ions react with the hydrogen ions in the tooth-biofilm interface by forming complexes, such as hydrogen phosphate and water. Thus saliva becomes undersaturated with respect to phosphate ions, which leads to the dissolution of hydroxyapettite crystals in an attempt to resaturate saliva.^20^

After a period of time, saliva saturates and mineral loss stops. When enough minerals (mainly calcium, phosphate, and hydroxyl ions) are available in the saliva surrounding the HA crystals, increasing its saturation level, balance returns.^22^ Thus, in our study, as the time interval increased the amount of calcium loss decreased.

Therefore, although pH is the strongest determinant for saturation level leading to demineralization or rem-ineralization under clinical conditions, but it is not the only important factor. Saturation is significantly affected by the calcium and phosphate ions concentration and the total ionic strength of plaque fluid.^22^

## CONCLUSION

Under the conditions of this study, following conclusions can be derived:

- Albumin has provided a strong protection against enamel demineralization at all times compared to the one without it.
- Since it is an *in vitro* study, further research should be carried out in *in vitro* environment for clinical application.
- Thus, albumin could be considered as an additive to oral hygiene products for caries-prone individuals with reduced salivary flow or altered saliva composition.
